# Succimer Chelation Improves Learning, Attention, and Arousal Regulation in Lead-Exposed Rats but Produces Lasting Cognitive Impairment in the Absence of Lead Exposure

**DOI:** 10.1289/ehp.9263

**Published:** 2006-10-30

**Authors:** Diane E. Stangle, Donald R. Smith, Stephane A. Beaudin, Myla S. Strawderman, David A. Levitsky, Barbara J. Strupp

**Affiliations:** 1 Department of Psychology, Cornell University, Ithaca, New York, USA; 2 Department of Environmental Toxicology, University of California, Santa Cruz, California, USA; 3 Division of Nutritional Sciences, Cornell University, Ithaca, New York, USA

**Keywords:** arousal regulation, attention, autism, behavior, chelation, delinquency, lead exposure, lead poisoning, learning, succimer, treatment

## Abstract

**Background:**

There is growing pressure for clinicians to prescribe chelation therapy at only slightly elevated blood lead levels. However, very few studies have evaluated whether chelation improves cognitive outcomes in Pb-exposed children, or whether these agents have adverse effects that may affect brain development in the absence of Pb exposure.

**Objectives:**

The present study was designed to answer these questions, using a rodent model of early childhood Pb exposure and treatment with succimer, a widely used chelating agent for the treatment of Pb poisoning.

**Results:**

Pb exposure produced lasting impairments in learning, attention, inhibitory control, and arousal regulation, paralleling the areas of dysfunction seen in Pb-exposed children. Succimer treatment of the Pb-exposed rats significantly improved learning, attention, and arousal regulation, although the efficacy of the treatment varied as a function of the Pb exposure level and the specific functional deficit. In contrast, succimer treatment of rats not previously exposed to Pb produced lasting and pervasive cognitive and affective dysfunction comparable in magnitude to that produced by the higher Pb exposure regimen.

**Conclusions:**

These are the first data, to our knowledge, to show that treatment with any chelating agent can alleviate cognitive deficits due to Pb exposure. These findings suggest that it may be possible to identify a succimer treatment protocol that improves cognitive outcomes in Pb-exposed children. However, they also suggest that succimer treatment should be strongly discouraged for children who do not have elevated tissue levels of Pb or other heavy metals.

Chelating agents have been used to treat lead-exposed children since the 1950s, but the metric for gauging therapeutic efficacy has changed over time. When these drugs were first implemented for clinical use, it was common for poor, inner-city children to be brought to emergency rooms with signs of Pb-induced encephalopathy. The chelating agents used at that time, CaNa_2_EDTA (calcium disodium versenate) and BAL (dimer-caprol), are credited with dramatically reducing the mortality rate in such children, estimated to be 66% before the advent of chelating agents (Foreman 1961) relative to 1–2% thereafter (Chisholm 1968). Fortunately, blood Pb levels of U.S. children have declined dramatically over the past 20 years [Centers for Disease Control and Prevention (CDC) 2005], and Pb-induced encephalopathy is rare. However, as evidence mounts that even slightly elevated Pb levels are associated with impaired cognitive functioning in children (e.g., Canfield et al. 2003), there appears to be growing pressure for clinicians to prescribe chelation therapy at very low exposure levels. The CDC (2005) and the American Academy of Pediatrics (2005) recommend chelation for blood Pb levels > 45 μg/dL, but surveillance data from 10 states indicate chelation therapy occurring at blood Pb levels well below this recommended level (CDC, unpublished data).

There are surprisingly little data upon which to gauge the benefits and possible risks of chelation therapy in children with subclinical Pb poisoning. With respect to succimer (dimercaptosuccinic acid), the most widely administered chelating agent, only one clinical trial conducted to date has included cognitive measures. This study did not detect a benefit of chelation, relative to placebo, in children with blood Pb levels between 20 and 44 μg/dL (Dietrich et al. 2004; Rogan et al. 2001). However, several studies using animal models reported that succimer normalized various behaviors altered by Pb exposure, including forced-swim immobility (Stewart et al. 1996), activity level, and habituation rate (Gong and Evans 1997).

Using a rodent model of early childhood Pb exposure, the present study was designed to *a*) determine whether treatment with a succimer regimen shown to produce significant reductions in blood and brain Pb levels (Cremin et al. 1999; Smith et al. 1998, 2000b; Stangle et al. 2004) also lessens the lasting cognitive and affective changes that are produced by a short period of early developmental Pb exposure; and *b*) determine whether succimer produces lasting cognitive and/or affective impairment when administered in the absence of Pb exposure. Findings from the latter group are needed to gauge the safety of prolonged regimens when treating Pb-exposed children, as well as the safety of the drug for treating autistic children, as currently advocated on numerous websites by physicians, autism parent groups, and organizations such as the American College for Advancement in Medicine.

In this article, we describe a subset of the administered tests—namely, a series of visual attention tests designed to tap several functions reported to be affected in Pb-exposed children, including sustained and selective attention, inhibitory control, learning/associative ability, and regulation of arousal or emotion. Two of these tasks, the sustained attention and selective attention tasks, are similar to ones commonly used to assess attention in human subjects, such as the Continuous Performance Test and Leonard’s 5-choice Serial Reaction Task (reviewed by Robbins 2002).

## Materials and Methods

### Study design

The study used a 3 × 2 factorial design, with three levels of Pb exposure and two levels of chelation (succimer or vehicle), described below. The sample size for the behavioral testing was 120 animals, 20 per group. These 120 animals were the female offspring of 60 litters, one succimer-treated and one vehicle-treated animal per litter. All procedures were approved by the Cornell Institutional Animal Care and Use Committee, and the housing facilities were accredited by the Association for Assessment and Accreditation of Laboratory Animal Care International.

### Breeding and Pb exposure

Nulliparous Long-Evans rats (Harlan Sprague Dawley, Indianapolis, IN) were mated in the laboratory. They were treated humanely and with regard for alleviation of suffering. Twenty-four hours after birth, each litter was culled to 10 pups and assigned to one of three Pb-exposure conditions [no Pb, moderate Pb (Mod-Pb), or high Pb (High-Pb)]. The dams in the no-Pb group received drinking water (deionized distilled) adulterated with 300 ppm sodium acetate from postnatal day (PND)1 to PND30. Both Pb-exposed groups received water adulterated with 300 ppm Pb acetate from PND1 to PND17. On PND17, around the age when pups begin consuming water directly from the drinking water bottle, the water Pb concentration was lowered to 20 ppm for the Mod-Pb group for the remainder of the Pb exposure period (until PND30) in order to maintain the moderate level of Pb intake experienced prior to PND17 when milk provided the sole source of Pb. The High-Pb group was maintained on the 300-ppm Pb acetate drinking water until PND30. At PND30, the pups were weaned and Pb exposure was terminated. The period of Pb exposure, PND1–30, corresponds roughly, in terms of neural development, to the period spanning the sixth month of pregnancy until late childhood/early adolescence in humans (Bayer et al. 1993; Rice and Barone 2000). Additional information on the Pb exposure regimens is provided in the Supplemental Material available online (http://www.ehponline.org/docs/2006/9263/suppl.pdf).

### Chelation

On PND30, the pups in each Pb treatment group were divided into succimer (Chemet; McNeil Consumer Products Company, Fort Washington, PA) and vehicle (apple juice) subgroups. This assignment was made at the level of each litter to provide littermate controls for the vehicle–succimer comparisons. Succimer or apple juice (vehicle) was administered twice daily via oral gavage from PND31 to PND52. The daily succimer dose was 50 mg/kg body weight/day during the first week of chelation, followed by 25 mg/kg/day for the second and third weeks, similar to the regimen used clinically (Rogan et al. 2001).

### Tissue Pb analyses

On PND52 (end of chelation therapy) one animal from each litter was euthanized for analysis of blood and whole brain Pb levels by inductively coupled plasma-high resolution mass spectrometry, as previously described (Smith et al. 2000b; see Supplemental Material available online at http://www.ehponline.org/docs/2006/9263/suppl.pdf).

### Testing apparatus

Behavioral testing was conducted in 12 automated Plexiglas chambers, each operated by a personal computer. Briefly, each chamber consisted of a waiting area, and a small testing alcove recessed into one wall. The alcove was separated from the waiting area by a metal guillotine-type door. Each of the three walls of the alcove contained a funnel-shaped response port. A light-emitting diode (LED) was mounted above each port; the illumination of one of these LEDs served as the discriminative cue in all of the tasks described here. For the selective attention task, compressed, scented air (which served as the olfactory distractors) was projected into the response ports via polyethylene tubes (see Gendle et al. 2003 for additional apparatus details).

### Testing procedures

Behavioral testing began on PND62. All animals were weighed and tested 6 days/week. For all tasks, a daily testing session consisted of 200 trials or 2 hr, whichever came first. All behavioral testing was conducted by individuals blind to the treatment condition of the subjects. Throughout behavioral testing, animals were maintained on a restricted food intake regimen (see Supplemental Material available online at http://www.ehponline.org/docs/2006/9263/suppl.pdf) with *ad libitum* access to water. Testing on the four tasks described in this report was initiated, on average, at 9, 10, 13, and 15 weeks of age, respectively.

### Visual discrimination task

Each trial began with the opening of the alcove door. Immediately after the animal broke the infrared beam at the alcove entrance, one of the three LEDs was illuminated. The LED remained illuminated until the animal made a 1-sec nose-poke into one of the three ports or 60 sec elapsed, whichever came first. A 1-sec nose-poke into the port under the illuminated LED constituted the correct response and was rewarded with delivery of a 45 mg food pellet into the alcove. Each animal was tested on this task until it reached a performance criterion of at least 80% correct in a single session. (For additional details of this task and the subsequent attention tasks, see Gendle et al. 2003, 2004).

### Visual discrimination with variable delay (attention task 1)

This task was a variation of the visual discrimination task with two modifications: *a*) a delay of varying duration (0, 3, 6, or 9 sec) was imposed between trial onset (entry into the alcove) and presentation of the cue (the illuminated LED); and *b*) the duration of cue illumination was shortened to 700 msec. Animals were tested on this task for 20 sessions.

### Sustained attention task

The sustained attention task was identical to the previous task, except that the duration of the cue illumination varied between 200, 400, and 700 msec. Animals were tested for 10 sessions on this task.

### Selective attention task

The animals were then tested for 10 sessions on a selective attention task, similar to the preceding task but with modifications of the stimulus parameters and the addition of unpredictable olfactory distractors on one-third or the trials in each daily test session. This task was preceded by three sessions on a baseline task, which had stimulus parameters identical to those in the selective attention task, but without olfactory distractors. In both tasks, the stimulus delay varied between 2 and 3 sec, and the stimulus duration varied between 300 and 700 msec.

### Statistical procedures

We analyzed the various performance measures using a generalized linear mixed model (GLMM), which correctly handled the repeated measures on each animal (Wolfinger and O’Connell 1993). Differences were considered to be significant at *p* < 0.05; *p*-values between 0.05 and 0.10 were considered to be trends and are discussed if they aid in clarifying the nature of the Pb and succimer effects. All statistical analyses were conducted using SAS 9.0 (SAS Institute, Cary, NC) for Windows 2000. Additional information on the various dependent measures and statistical analyses is provided in the Supplemental Material available online (http://www.ehponline.org/docs/2006/9263/suppl.pdf).

## Results

Because of space constraints, the results of behavioral testing presented here include only those instances where effects of Pb and/or succimer were seen.

### Comparison of the High-Pb, High-Pb–Succimer, and Control Groups

#### Visual discrimination task

Analysis of average percent errors for the first six sessions on the visual discrimination task revealed a significant main effect of treatment group [F_(2, 78.1)_ = 8.54; *p* = 0.0004] and a significant interaction of treatment and session number [F_(10, 151)_ = 3.29; *p* = 0.0007]. The controls (no lead exposure and no succimer treatment) performed significantly better than the High-Pb (*p* = 0.017) and High-Pb–succimer (*p* < 0.0001) groups. Chelation offered no benefit; the performance of the High-Pb and High-Pb–succimer groups did not differ significantly. The High-Pb and control groups did not differ significantly by the sixth session, indicating that their inferior performance earlier in testing reflected impaired learning ability.

#### Visual discrimination with variable delay (attention task 1)

##### Percent premature responses

**Figure 1:**
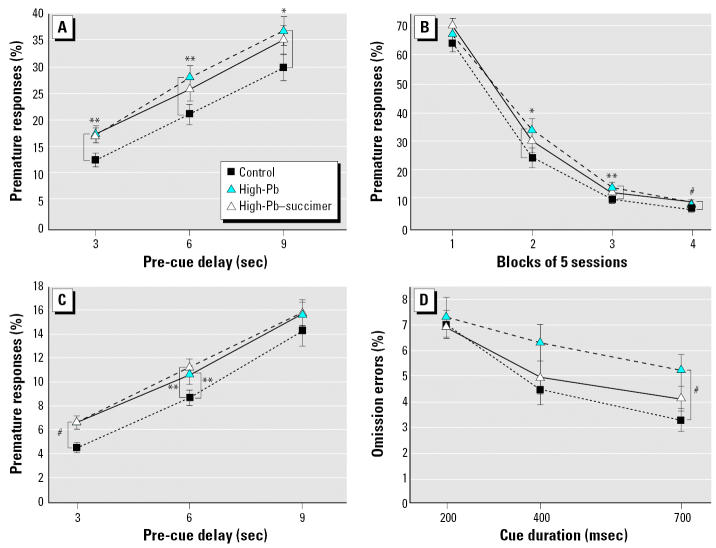
Succimer treatment of the High-Pb rats was either ineffective or only partially effective in alleviating the impairments in learning, inhibitory control, and early-session attentional function. Percent premature responses in attention task 1 as a function of (*A*) the duration of the pre-cue delay (averaged across the 20 sessions) and (*B*) stage of testing (averaged across the pre-cue delay). (*C*) Percent premature responses in the sustained attention task as a function of the duration of the delay between trial onset and cue presentation. (*D*) Percent omission errors committed during the first block of trials in each session (trials 1–66) in the sustained attention task as a function of cue duration (slopes for High-Pb vs. control; *p* = 0.009). Data points are means ± SEs. *p ≤ 0.07. **p < 0.05. ^#^*p* ≤ 0.01.

Analysis of percent premature responses (responses made before cue onset) revealed a borderline main effect of treatment [F_(2, 57.2)_ = 2.81; *p* = 0.068]; and significant interactions involving treatment and delay [F_(4, 1925)_ = 3.64; *p* = 0.006], and treatment, session block, and trial block [F_(12, 1926)_ = 2.13; *p* = 0.013]. Rats in the High-Pb group committed a significantly higher percentage of premature responses than controls for trials with the 3 and 6 sec pre-cue delays (averaged across the 20 sessions on the task) (Figure 1A), and for session blocks 3 and 4 (averaged across pre-cue delay) (Figure 1B). Similar trends were seen for trials with a 9-sec pre-cue delay (Figure 1A) and for session block 2 (Figure 1B). The extent to which succimer treatment improved performance varied across conditions. Under some conditions (e.g., session blocks 2 and 3), the succimer treatment offered some benefit, as the performance of the High-Pb–succimer group was intermediate to the control and High-Pb groups, not differing from either one (Figure 1B). In other conditions [e.g., pre-cue delays 3 and 6 (Figure 1A); session block 4 (Figure 1B)], no benefit was seen; that is, rats in the High-Pb–succimer group committed a significantly higher percentage of premature responses than the controls and did not differ from the High-Pb group.

##### Alcove latency

**Figure 2:**
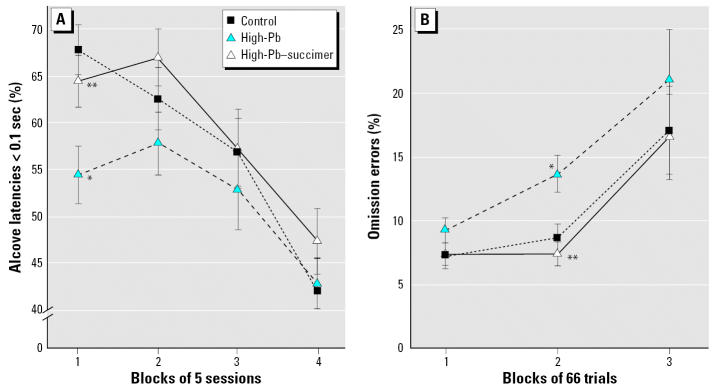
Heightened reactivity to errors of the High-Pb rats was completely normalized by succimer treatment. (*A*) The percentage of trials in attention task 1 for which the latency to enter the testing alcove at trial onset was very short (< 0.1 sec) across the 4 blocks of sessions (20 sessions). (*B*) Percent omission errors for trials following an error in the sustained attention task, plotted as a function of the block of trials within each 200-trial testing session (averaged across the 10 sessions). Data points are means ± SEs. *p < 0.01, High-Pb vs. control. **p < 0.01, High-Pb–succimer vs. High-Pb.

Alcove latency (AL) is the time elapsed between the raising of the alcove door at trial onset and entry of the animal into the testing alcove. Because the data were highly skewed, with a high proportion of identical values (the minimum latency), the dependent measures used for each animal were the percentages of ALs that fell into each of four bins of latency values: < 0.1, 0.1–0.5, 0.6–1.0, and > 1 sec. A significant treatment × session block interaction was found in the analysis of very short ALS [F_(6, 52.2)_ = 3.24; *p* = 0.009]. As seen in Figure 2A, the percentage of very short ALs (< 0.1 sec) was significantly lower for the High-Pb rats than for controls specifically during the first block of sessions (*p* = 0.001), suggesting some hesitation to enter the testing alcove during this early portion of the task when all groups were performing very poorly. Succimer treatment was beneficial in that the High-Pb–succimer rats did not exhibit this hesitancy in the first block of sessions: they did not differ from controls (*p* = 0.38) and differed significantly from the High-Pb group (*p* = 0.01).

##### Response latency

We defined response latency as the time between cue onset and a response. The data were log-transformed before analysis. Analysis of the High-Pb, High-Pb–succimer, and control groups revealed significant interactions of treatment and delay [F_(6, 2700)_ = 2.70; *p* = 0.013], and treatment, delay, and session block [F_(18, 2700)_ = 1.85; *p* = 0.016]. The increased response latency seen early in the task for all animals (when error rate was very high) was more pronounced for the High-Pb rats than for the control and High-Pb–succimer groups. This Pb effect was normalized by succimer treatment: the High-Pb–succimer group was significantly faster than the High-Pb group during these early sessions and did not differ from controls (i.e., the same pattern seen for AL, described above).

#### Sustained attention task

##### Percent premature responses

Analysis of premature response rate for the High-Pb, High-Pb–succimer, and control groups revealed significant effects of treatment [F_(2, 58.9)_ = 3.56; *p* = 0.035] and treatment × delay [F_(4, 114)_ = 2.81; *p* = 0.029]. The High-Pb group committed a higher percentage of premature responses than the controls for trials with a 3-sec (*p* = 0.001) or 6-sec (*p* = 0.05) pre-cue delay (Figure 1C). Succimer treatment did not improve this deficiency in inhibitory control; the High-Pb–succimer group was significantly different from controls for the 3-sec (*p* = 0.001) and 6-sec (*p* = 0.02) delays and was not different from the High-Pb group for either.

##### Percent omission errors

An omission error was tallied when a rat entered the testing alcove but failed to respond within 15 sec of trial onset, indicative of missing the cue. The analysis of percent omission errors for the High-Pb, High-Pb–succimer, and control groups revealed the following significant treatment-related effects: treatment × trial block [F_(4, 107)_ = 2.91; *p* = 0.025], treatment × trial block × cue duration [F_(12, 872)_ = 1.75; *p* = 0.05], and treatment × trial block × previous trial outcome (Prev) [F_(4, 892)_ = 6.63; *p* < 0.0001]. For all groups, we found an increase in percent omission errors across each session, an effect that was most pronounced for trials that followed an error. As shown in Figure 2B, for trials following an error, the rate of increase in omission errors across each session was greater for the High-Pb rats than for controls. This impairment was abolished by succimer treatment: The High-Pb–succimer group was significantly different from the High-Pb-group and did not differ from controls.

The significant interaction of treatment, trial block, and cue duration [F_(12, 872)_ = 1.75; *p* = 0.05] reflected that *a*) the High-Pb rats committed a borderline greater percent omission errors than controls in the first block of trials in each session (*p* = 0.06), and *b*) during this first block of trials in each session (trials 1–66), the High-Pb group benefited less than controls by the lengthening of the cue from 200 to 700 msec (*p* = 0.009), such that group differences were greatest on trials with the longest cue duration (*p* = 0.01, for 700 msec cue; Figure 1D). Succimer treatment provided some benefit, as the High-Pb–succimer group did not differ significantly from controls in this regard; however, they also did not differ significantly from the High-Pb group.

Additional details of the omission error analyses are provided in the Supplemental Material available online (http://www.ehponline.org/docs/2006/9263/suppl.pdf).

### Comparison of the Mod-Pb, Mod-Pb–Succimer, and Control Groups

#### Visual discrimination task

**Figure 3:**
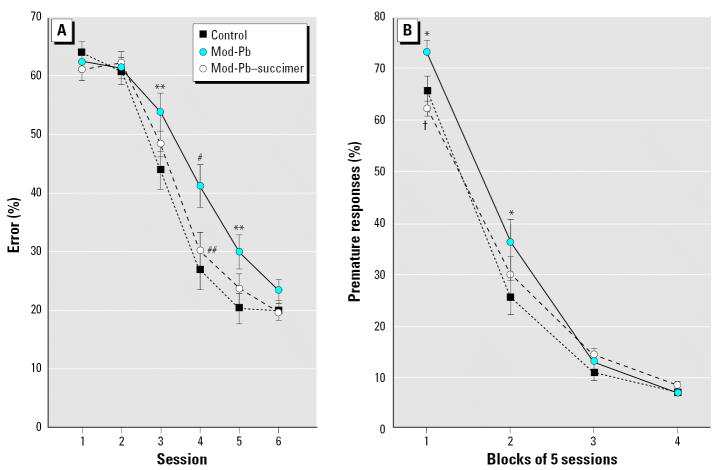
Succimer treatment significantly improved learning ability of the Mod-Pb rats. (*A*) Visual discrimination task (Mod-Pb–succimer vs. Mod-Pb; main effect contrast, *p* = 0.03). (*B*) Attention task 1. Data points are means ± SEs. *p = 0.056; **p ≤ 0.03; ^#^*p* < 0.01, Mod-Pb vs. control. ^##^*p* = 0.03; †*p* = 0.006, Mod-Pb–succimer vs. Mod-Pb.

Analysis of average percent errors for the first six sessions on the task revealed a significant main effect of treatment group (F_(2, 78.1)_ = 4.88; *p* = 0.01) and a significant interaction of treatment and session [F_(10, 159)_ = 1.93; *p* = 0.04]. The Mod-Pb group learned significantly more slowly than controls (main effect contrast, *p* = 0.004). Chelation offered a significant benefit in that the Mod-Pb–succimer group learned significantly faster than the Mod-Pb group (main effect contrast, *p* = 0.03) and did not differ from controls for any session. The three groups performed similarly by the sixth session, indicating that the observed group differences in performance reflected variations in learning ability (Figure 3A).

#### Visual discrimination with variable delay (attention task 1)

The analysis of percent premature responses revealed a significant interaction of treatment and session block [F_(6, 58.9)_ = 2.89; *p* = 0.016]. As depicted in Figure 3B, the Mod-Pb group mastered the new rule (i.e., that the light cue was presented after a delay on some trials) more slowly than controls (*p* = 0.05), and succimer treatment offered a significant benefit (Mod-Pb vs. Mod-Pb–succimer, session block 1, *p* = 0.006). The Mod-Pb–succimer and control groups did not differ for any session. The performance of the three groups did not differ during the final 10 sessions on the task (blocks 3 and 4), indicating that the observed performance differences reflected differing learning ability.

### Comparison of the Succimer-Only and Control Groups

#### Visual discrimination task

**Figure 4:**
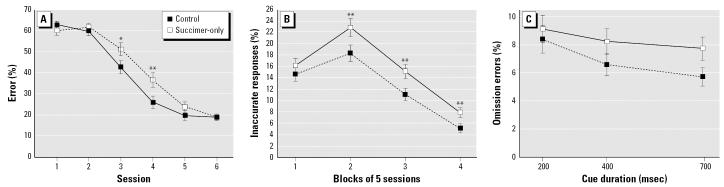
Succimer treatment of the non-Pb–exposed rats impaired performance in (*A*) visual discrimination task (main effect contrast, *p* = 0.04), (*B*) attention task 1, and (*C*) the sustained attention task (treatment × cue duration, *p* = 0.004). Data points are means ± SEs. *p = 0.07; **p < 0.05, succimer-only vs. controls.

Analysis of average percent errors for the first six sessions on the task revealed that the succimer-only group (treated with succimer in the absence of lead exposure) learned significantly more slowly than the controls [Figure 4A; treatment (F_(1, 57)_ = 4.21; *p* = 0.04); treatment × session (F_(5, 99.4)_ = 2.20; *p* = 0.06)].

#### Attention task 1

The percentage of inaccurate responses (responses made after cue onset, but to an incorrect port) was significantly higher for the succimer-only group than for controls during session blocks 2, 3, and 4 [Figure 4B; treatment, (F_(1, 141)_ = 4.42; *p* = 0.042); treatment × session block (F_(3, 1729)_ = 2.64; *p* = 0.048)]. The groups did not differ significantly for this type of error during session block 1 (sessions 1–5), an early point in the learning process when most responses were made before cue onset (premature responses).

#### Sustained attention task

##### Percent omission errors

The analysis of omission errors for the control and succimer-only groups revealed significant interactions of treatment and cue duration [F_(2, 587)_ = 4.47; *p* = 0.012] and of treatment, cue duration, and trial block [F_(8, 587)_ = 1.99; *p* = 0.045]. As shown in Figure 4C, the succimer-only group benefited less than controls by the lengthening of the cue from 200 to 700 msec (*p* = 0.004). This pattern was seen only during the beginning and end of each daily session (contrasts for slopes across cue duration, *p* = 0.002 and 0.037 for first and third trial blocks, respectively).

##### Percent inaccurate responses

The succimer-only group benefited less than controls from the lengthening of the cue, resulting in increasing group differences in percent inaccurate responses as the cue duration increased [F_(2, 1292)_ = 15.95; *p* < 0.0001].

#### Selective attention task

Because the timing of the distractor relative to cue onset (1 or 2 sec) did not alter the treatment-related effects, we used a two-level variable for distraction condition for the analyses: distractor or no distractor.

**Figure 5:**
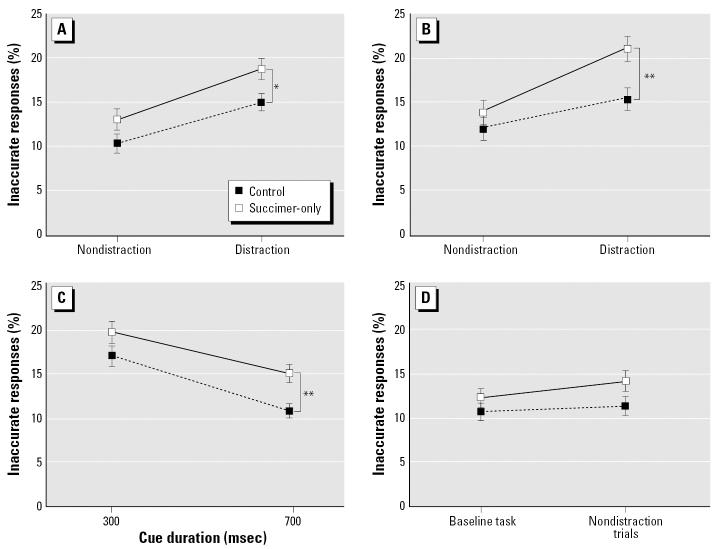
Succimer treatment of the non-Pb-exposed rats impaired performance in the selective attention task. Percent inaccurate responses in the selective attention task, as a function of whether or not a dis-tractor was presented on the current trial and whether the prior trial was correct (*A*) or incorrect (*B*). (*C*) Percent inaccurate responses in the selective attention task, plotted as a function of cue duration. (*D*) Percent inaccurate responses in the baseline task and the nondistraction trials of the selective attention task (see text). Data shown are means ± SEs. *p = 0.02; **p < 0.01, succimer-only vs. controls.

The analysis of percent inaccurate responses for the succimer-only and control groups revealed a main effect of treatment group [F_(1, 38.1)_ = 6.18; *p* = 0.017], an interaction of treatment and cue duration [F_(1, 216)_ = 11.25; *p* = 0.0009], and a trend toward a three-way interaction of treatment, distraction condition, and the outcome of the previous trial [F_(1, 387)_ = 3.06, *p* = 0.08]. The succimer-only group benefited less by the lengthening of the cue than did controls (Figure 5C). In addition, although the succimer-only group committed a higher percentage of inaccurate responses overall (*p* = 0.017), group differences were greatest for trials that both included a distractor and that followed an error (Figure 5A,B). However, there was also a tendency for the groups to differ on non-distraction trials (*p* = 0.10; Figure 5A). To assess whether the trend toward poorer performance of the succimer-only group on the nondistraction trials reflected generalized disruption caused by the unpredictable presentation of the olfactory distractors, an analysis was conducted to directly compare performance on the nondistraction trials with that of the preceding baseline task. All trials in this latter task were identical to the nondistraction trials of the selective attention task (i.e., pre-cue delays, cue durations, etc.). For the succimer-only group, the percentage of inaccurate responses was significantly higher for the non-distraction trials of the selective attention task than during the baseline task (*p* = 0.0001), whereas for controls, the rates were similar for these two conditions (*p* = 0.16; Figure 5D). This analysis supports the inference that for the succimer-only group, the unpredictable presentation of the olfactory distractors produced a generalized disruption in performance that extended beyond the distraction trials.

##### Motivation

Motivation was assessed by analyzing the average rate of response trials [number of response trials per session divided by session length (minutes)]. No significant group differences were detected (all *p* > 0.2), providing evidence that motivational differences did not account for the observed differences in performance (for further discussion, see Supplemental Material available online at http://www.ehponline.org/docs/2006/9263/suppl.pdf).

##### Blood and brain Pb levels

Analysis of mean blood Pb levels on PND52 revealed dose-dependent effects on both blood and brain Pb levels and a beneficial effect of succimer chelation for both Pb exposure conditions for both tissues (Table 1).

##### Body weight and general health

The High-Pb and High-Pb–succimer groups weighed ≈6% less than the control group (*p* < 0.05), measured on PND52 after cessation of succimer treatment. None of the other groups differed from controls. The slightly smaller size of the High-Pb rats likely reflects a decrease in growth hormone during the Pb exposure period (e.g., Ronis et al. 1998; see Supplemental Material available online at http://www.ehponline.org/docs/2006/9263/suppl.pdf). Low-level Pb exposure in children has also been associated with small but statistically significant reductions in height, in the absence of any other signs of overt toxicity (e.g., Shukla et al. 1991).

## Discussion

A short period of early Pb exposure produced impairments in learning, attention, inhibitory control, and arousal regulation, paralleling the areas of dysfunction seen in children with low to moderate elevations in blood Pb levels. As in children, these impairments were lasting; that is, they were apparent long past the period of Pb exposure, indicative of lasting neurologic dysfunction as a result of the early exposure. The pervasiveness of the impairment was related to the intensity of Pb exposure; for the Mod-Pb group, dysfunction was limited to learning ability, whereas the High-Pb group exhibited impairments in learning, inhibitory control, arousal regulation, and attention. Importantly, succimer treatment significantly alleviated some Pb-induced neurobehavioral deficits, although the magnitude of the benefit varied as a function of both the level of the Pb exposure and the specific area of dysfunction. In contrast, succimer treatment of rats not previously exposed to Pb produced lasting cognitive and affective dysfunction, similar in magnitude and pervasiveness to that produced by the High-Pb exposure regimen. The basis for each of these conclusions is provided below and summarized in Table 2.

### Pb-induced learning deficits and efficacy of succimer treatment

Both the High-Pb and Mod-Pb groups learned the basic rules of the visual discrimination task and attention task 1 more slowly than the controls, indicating lasting impairment in associative ability as a result of a short period of early Pb exposure, as previously reported (e.g., Garavan et al. 2000). Succimer treatment of the Pb-exposed animals improved learning rate, although the degree of benefit was greater for the Mod-Pb group than for the High-Pb group. Succimer treatment significantly improved the learning rate of the rats in the Mod-Pb group for both the initial visual discrimination task and attention task 1; in both tasks, the Mod-Pb–succimer group learned significantly faster than the Mod-Pb group and did not differ significantly from the controls. For the High-Pb exposure regimen, succimer treatment did not improve learning rate in the visual discrimination task, but a small benefit was seen in attention task 1. It is likely that more prolonged succimer treatment would have produced an even greater cognitive benefit for these more heavily exposed animals. This suggestion is based on the facts that *a*) reductions in brain Pb levels lag significantly behind reductions in blood Pb following chelation or cessation of Pb exposure (Cremin et al. 1999; Smith et al. 1998; Stangle et al. 2004); *b*) blood and brain Pb levels were still quite elevated in the High-Pb group at the end of chelation (approximately equal to the nonchelated Mod-Pb rats); and *c*) in a previous study from our laboratory, a second 3-week succimer regimen offered significant benefit over one regimen in terms of both blood and brain Pb reductions (Stangle et al. 2004). Therefore, these results likely underestimate the potential benefit of succimer treatment for the more heavily Pb-exposed animals.

### Heightened reaction to errors in the Pb-exposed rats is abolished by succimer treatment

For the four tasks presented here, the rate of all types of errors was significantly greater on trials that followed an error than on trials following a correct response. Similarly, the latency to enter the testing alcove at trial onset was significantly longer on trials following an error than on trials that followed a correct response, a pattern also seen for the latency to make a response after cue onset. The disruption produced by committing an error was significantly greater for the High-Pb animals than for controls for several dependent measures: In the sustained attention task, the percentage of omission errors was significantly higher for the High-Pb group than for controls during mid- to late-session trials following an error—but not following a correct response—a pattern that indicates the combined influences of the disruptive effects of committing an error and the changing motivational and attentional state of the animals across each session. Similarly, in attention task 1, the High-Pb rats took significantly longer to enter the testing alcove and to make a response than controls, but only early in the task when error rate was very high and the rats had not yet learned the task rules. Finally, the drop in performance on trials following an error was also more pronounced for the High-Pb rats than for controls in a conditional olfactory discrimination task with periodic reward omission, an additional task administered subsequently to these rats (Beaudin et al. 2006).

The interpretation of the heightened sensitivity to errors of the High-Pb rats is informed by previous studies that examined performance changes as a function of an error on the previous trial, all involving human subjects. In some of these studies, the error rate on post-error trials was exceptionally low (e.g., Laming 1979; Robertson et al. 1997), indicating the operation of an executive error-correction system localized to the anterior cingulate cortex (e.g., Bush et al. 2000; Fernandez-Duque et al. 2000). However, the finding consistently seen in our rodent studies—increased error rate on trials following an error (reviewed by Strupp and Beaudin 2006)—has also been reported in some human studies (e.g., Elliott et al. 1996; Rabbitt and Rodgers 1977) and likely reflects an emotional response to the error. This interpretation is supported by the finding that an electrophysiologic marker of error detection, termed error-related negativity, varies as a function of individual differences in negative affect and emotionality (e.g., Luu et al. 2000; also see Beaudin et al. 2006).

Importantly, this heightened disruption following an error was very responsive to succimer treatment: In all cases, the High-Pb–succimer group was indistinguishable from controls (Figure 2; Beaudin et al. 2006). These results provide encouragement that Pb-induced dysfunction in the area of arousal or emotion regulation can be significantly alleviated by succimer treatment. This type of dysfunction may be an important contributor of the behavioral problems and increased delinquency rates seen in Pb-exposed children and adolescents (e.g., Bellinger et al. 1994; Dietrich et al. 2001; Needleman et al. 1996), based on evidence that individuals vulnerable to faulty regulation of negative emotion are at risk for violence and aggression (Davidson et al. 2000).

### Succimer was effective in alleviating some, but not all, types of Pb-induced attentional dysfunction

The sustained attention task revealed two types of attentional dysfunction in the High-Pb group. First, the early-session increase in omission errors (relative to mid-session), seen for all groups across all sessions on this task, was more pronounced for the High-Pb group than for the controls, with group differences being largest for trials with the longest cue duration (700 msec). This pattern of results may indicate that lapses in attention were more common for the High-Pb rats than for controls during this early part of the session. More frequent attentional lapses in the High-Pb group would have the consequence of flattening the slope across cue duration and making group differences largest on trials for which performance of the controls was best (i.e., those with the longest cues). One interpretation of this pattern is that early Pb exposure may impair the ability to rapidly engage in a new task when transitioning between activities, manifested here as an increased tendency to miss the cue (an attentional deficit).

A second type of attentional dysfunction observed in the High-Pb rats was evident later in each testing session. The incidence of omission errors increased for all groups across each daily testing session, the classic pattern seen when sustained attention is taxed (Parasuraman and Warm 1998). However, as discussed above, the High-Pb rats committed a significantly higher percentage of omission errors than controls during mid- to late-session trials, specifically on trials that followed an error. This pattern of effects, coupled with the similar pattern seen in the omission of reward task administered subsequently to these same animals (Beaudin et al. 2006), indicates that as attention and motivation waned across the session, the disruptive effect of an error on attention and persistence increased, and that this disruption was more pronounced for the Pb-exposed rats than for controls.

The effectiveness of succimer chelation varied for these different types of attentional deficits. As discussed above, the heightened attentional disruption seen in the High-Pb rats following an error was completely normalized by succimer treatment, as the chelated High-Pb rats differed significantly from the High-Pb group and did not differ from controls. In contrast, succimer treatment only partially alleviated the attentional lapses seen early in each test session; the High-Pb–succimer group was intermediate to the other two, not differing from either.

### Deficient inhibitory control in the High-Pb rats

In the sustained attention task, the percentage of premature responses was significantly higher for the High-Pb rats than for controls, indicating deficient inhibitory control. Converging evidence for this area of dysfunction was provided by the higher percentage of premature responses committed by the High-Pb rats in the final block of trials of attention task 1, a point at which the basic rules of the task had already been learned. Succimer treatment was ineffective in alleviating this area of dysfunction: In both instances, the percentage of premature responses for the High-Pb–succimer rats was significantly greater than that of the control rats, and not different from the High-Pb group.

### Pattern of succimer effects in the Pb-exposed animals

The efficacy of the succimer treatment varied as a function of both the level of Pb exposure and the specific functional domain tested. Interestingly, the pattern seen for succimer efficacy parallels the apparent sensitivity of specific functional domains to the Pb exposure. Learning was impaired by both Pb exposure regimens, whereas impaired regulation of arousal or emotion was seen only in the High-Pb group, suggesting that disruption in learning occurs at lower exposures than dysregulation of arousal or emotion. The efficacy of succimer across these domains corresponded to the degree to which chelation reduced brain Pb levels in the two Pb exposure groups. For the Mod-Pb group, succimer treatment almost completely removed Pb from the brain and effectively alleviated the learning dysfunction. In contrast, brain Pb levels were still moderately elevated in the High-Pb group following chelation; these chelated High-Pb rats exhibited the same pattern of dysfunction as the unchelated Mod-Pb group, which also had moderately elevated brain Pb levels on PND52. Both groups also exhibited impaired learning but not affective dysfunction. This constellation of findings suggests that succimer treatment of the High-Pb rats reduced tissue (i.e., brain) Pb levels below that which produces lasting impairment in emotion regulation, but not enough to alleviate the learning dysfunction.

The mechanistic basis for this pattern of effects is unknown, but likely reflects complex interactions between the timing of the Pb exposure and succimer treatment relative to the ontogenetic stage of specific neural systems, the extent to which succimer treatment reduced Pb levels in specific neural systems (Cremin et al. 1999), and the inherent sensitivity of those developmental processes and/or associated functional domains to Pb exposure.

### Effects of succimer in the absence of Pb exposure

The present study also revealed the unexpected finding that a single 3-week course of succimer treatment during early development produced lasting dysfunction in cognition and arousal regulation in rats not previously exposed to Pb. Note that these four behavioral tests were administered across a 7-month period following cessation of succimer treatment, suggesting lasting brain changes. These results corroborate preliminary results from a similar study in nonhuman primates (Laughlin 2001). The succimer-only group learned the initial visual discrimination task more slowly than the controls. They also committed a higher rate of inaccurate responses than controls in attention task 1, indicative of attentional dysfunction. In both the sustained attention task and the selective attention task, their performance benefited less by the lengthening of the visual cue than did the controls, indicative of lapses in attention, as discussed above. Additionally, in the selective attention task, the succimer-only group committed a higher rate of inaccurate responses than controls, an effect that was most pronounced for trials on which a distractor was presented and that followed an error, indicating impairments in both selective attention and arousal regulation. Finally, a comparison of performance on trials without distractors (during the selective attention task) to performance on the baseline task revealed that the succimer-only rats experienced a generalized disruption of performance in the selective attention task that extended beyond the trials on which a distractor was presented. This finding, too, implicates impaired arousal regulation in the succimer-only group. These various impairments were similar in magnitude to those produced by the High-Pb exposure.

The mechanism(s) responsible for these adverse succimer effects is not known. One possibility is that succimer, a metal-chelating agent, altered essential metal homeostasis and increased metal diuresis, as has been suggested by a number of clinical and nonhuman primate studies (Aposhian et al. 1989; Fournier et al. 1988; Smith et al. 2000a).

### Extrapolation of treatment regimens to humans

Both Pb-exposed groups exhibited low blood Pb levels (≈25–35 μg/dL) during the first 3 weeks of the 4-week exposure period. During the fourth and final week of exposure, both groups experienced an increase in tissue Pb levels because of the direct ingestion of Pb-adulterated water. Although the blood Pb levels produced during this final week of exposure were higher than commonly reported for children (see Supplemental materials available online at http://www.ehponline.org/docs/2006/9263/suppl.pdf), direct comparison of blood Pb levels across species should be cautioned. The available evidence indicates that the blood Pb levels required to produce overt toxicity (i.e., malaise, coma, convulsions, death) or lasting neurobehavioral effects (i.e., effects that last beyond the period of exposure) are higher in both rats and nonhuman primates than in humans (Garavan et al. 2000; Levin and Bowman 1986; Morgan et al. 2001; Rice 1988). Consistent with these earlier studies, none of the animals in the present study exhibited any signs of overt toxicity throughout the study and could not be distinguished from controls throughout the 8 months of daily handling. The Mod-Pb group exhibited very circumscribed and subtle behavioral/cognitive effects, and the High-Pb group exhibited subtle, although more widespread, neurobehavioral effects. As such, the regimens used in the present study may be viewed as modeling subclinical or asymptomatic Pb exposure. Similarly, although the succimer regimen used in the present study closely modeled the duration and dose (weight-normalized) of the regimens used clinically in children, it is possible that it was more “aggressive” than the clinical regimen, when allometrically scaled across species.

## Summary and Implications

The present study provides clear evidence that administration of succimer, using a regimen sufficient to reduce brain Pb levels, can lessen Pb-induced impairments in learning ability, attention, and regulation of arousal and/or emotion. These are the first data, to our knowledge, to show that treatment with any chelating agent can alleviate cognitive deficits caused by Pb exposure. The primary area of dysfunction seen in the Mod-Pb rats, impaired learning ability, was significantly alleviated by succimer treatment. For the High-Pb group, treatment with succimer provided a robust benefit on measures indicative of impaired regulation of arousal or affect—one of the most pervasive areas of impairment seen in this group, and a major cause of their impaired performance overall. However, for the High-Pb group, succimer produced only a slight improvement in learning ability and did not lessen the deficient inhibitory control. The High-Pb rats treated with succimer performed similarly to non-chelated Mod-Pb rats—consistent with their comparable brain Pb levels—suggesting that a more prolonged treatment may have further improved performance in the High-Pb exposed animals.

These findings are consistent with previous animal studies but contrast with the results of the Treatment of Lead-Exposed Children (TLC) study, the one clinical trial in Pb-exposed children that included cognitive outcomes (Dietrich et al. 2004; Rogan et al. 2001). There are several possible reasons for this disparity in outcomes. First, the succimer treatment protocol used in the TLC trial may not have achieved a sufficient reduction in brain Pb levels to improve cognitive functioning. This suggestion is based on the evidence that *a*) succimer-induced reductions in blood Pb overestimate reductions in brain lead (Cremin et al. 1999; Smith et al. 1998; Stangle et al. 2004) and *b*) the succimer treatment protocol used in the TLC study achieved relatively modest group differences in blood Pb levels (relative to placebo), averaging only 4.5 μg/dL over the 6 months following treatment (Rogan et al. 2001). In comparison, in the present study both blood and brain Pb levels were substantially reduced by succimer, relative to the vehicle treatment (Table 1). Second, the disparate outcomes of the TLC trial and the present study may also reflect differences in the nature of the cognitive tasks that were used. In particular, the tasks used here (relative to those in the TLC trial) may have provided more specific indices of the two functional domains most improved by succimer in the present study: associative learning ability and regulation of arousal and/or emotion (indexed by reactivity to errors).

In conclusion, this study demonstrates that it is possible for chelation therapy to alleviate certain types of Pb-induced behavioral/cognitive dysfunction in rats. This is an important demonstration because Pb-induced behavioral dysfunction is generally considered to be irreversible (e.g., Needleman et al. 1996). The present findings thus suggest that if a succimer treatment protocol that produced a substantial removal of Pb from the brain could be identified for humans, a functional benefit might be derived. In addition, the finding that succimer produced lasting adverse effects when administered to non-Pb–exposed rats highlights the potential risks of administering succimer or other metal chelating agents to children who do not have elevated tissue Pb levels. It is of significant concern that this type of therapy is being widely advocated as safe and effective for treating autism.

## Correction

In the original manuscript published online, the symbols for the control and succimer-only groups were reversed in Figure 4A. They have been corrected here.

## Figures and Tables

**Table 1:** Mean ± SE blood and brain Pb levels at PND52 (end of succimer treatment).

Group	Blood Pb (μg/dL)	Brain Pb (ng/g dw)^a^
Control	1.5 ± 0.1	41 ± 9
Mod-Pb	12.6 ± 0.8*	1,040 ± 49*
High-Pb	31.0 ± 0.8**	3,690 ± 260**
Mod-Pb–succimer	2.8 ± 0.2#	196 ± 14.2#
High-Pb–succimer	8.5 ± 0.7##	1,370 ± 150##

*n* = 7–11 animals per group.

a ng Pb/g dry tissue.

* *p* < 0.005;

** *p* < 0.0001, Pb-exposed (nonchelated) groups vs. control.

# *p* < 0.003 for both blood and brain, Mod-Pb–succimer vs. Mod-Pb.

## *p* < 0.0001 for both blood and brain, High-Pb–succimer vs. High-Pb.

**Table 2:** Each of the areas of impairment seen in the High-Pb, Mod-Pb, and succimer-only groups, followed by the specific finding upon which the functional inference is based.

Area of dysfunction (specific measure)	Alleviated by succimer?
High-Pb group	
Impaired learning ability	
Visual discrimination task: rate of decline of percent errors	N
Attention task 1: rate of decline of percent premature responses	I
Impaired Inhibitory control	
Attention task 1: increased percent premature responses in final block of sessions	N
Sustained attention task: percent premature responses (throughout all sessions)	N
Increased disruption following errors (impaired arousal regulation)	
Attention task 1: increased alcove latency in first block of sessions	Y
Attention task 1: increased response latency in first block of sessions	Y
Sustained attention task: increased omission errors on post-error trials	Y
Lapses in attention (pattern of group differences as a function of cue duration)	I
Early session increase in omission errors (transition problems → attention dysfunction)	I
Mod-Pb Group	
Impaired learning ability	
Visual discrimination task: rate of decline of percent errors	Y
Attention task 1: rate of decline of percent premature responses	Y
Succimer-only group	
Impaired learning ability: visual discrimination task (rate of decline of percent errors)	
Attentional dysfunction: attention task 1 (increased percent inaccurate responses)	
Lapses in attention (pattern of effects as a function of cue duration)	
Sustained attention task	
Selective attention task	
Impaired arousal regulation	
Selective attention task: group differences greatest on post-error distraction trials	
Higher rate of errors on nondistraction trials in selective attention task than in baseline task	

For the High-Pb and Mod-Pb groups, the right-hand column indicates whether succimer treatment significantly alleviated the dysfunction (Y), did not significantly alleviate dysfunction (N), or produced a level of performance that was intermediate (I; i.e., not significantly different from either the control group or their vehicle-treated counterparts).
