# Supplementary material for: Succimer Chelation Improves Learning, Attention, and Arousal Regulation in Lead-Exposed Rats but Produces Lasting Cognitive Impairment in the Absence of Lead Exposure
Source: Environ Health Perspect. 2006 Oct 30;115(2):201–9. doi: 10.1289/ehp.9263 (PMC1831518; doi:10.1289/ehp.9263)
Supplement: Supplemental Figures and Tables [file ehp0115-000201s1.pdf]

Supplemental material

Lead exposure regimen: Both lead-exposed groups received water adulterated with 300ppm lead acetate from PND 1-17, a regimen which produces blood lead levels of approximately 40 µg/dL in the dams (e.g., Alber and Strupp 1996). Based on many prior studies in our lab using this regimen, the blood lead levels of the pups during PND 1-17, when the dams' milk provides the sole source of lead, is ≈ 25-35 µg/dL (Garavan et al. 2000; Hilson and Strupp 1996; Morgan et al. 2001; Smith et al. 1998). At PND 17, the age at which pups generally begin to drink water from the bottle in the cage, the concentration of lead acetate in the drinking water was lowered to 20 ppm for one of the two lead-exposed groups, termed the Moderate Pb (Mod Pb) group, for the remainder of the lead exposure period (until PND 30). With this regimen, the average blood lead level by the end of treatment is ≈ 40-60 µg/dL (Stangle et al. 2004; Strupp et al. unpublished observations). The other lead exposure group, termed the High Pb group continued to receive water adulterated with 300 ppm lead acetate. Blood leads for this group average ≈ 100-140 µg/dL by PND 30 and then rapidly decline (Garavan et al. 2000; Morgan et al. 2001; Smith et al. 1998; Stangle et al. 2004; Strupp et al. unpublished data). This exposure regimen was included here to ensure detection of lead-induced cognitive dysfunction, as we have demonstrated cognitive deficits with this regimen in prior studies (e.g., Garavan et al. 2000; Morgan et al. 2001). Although reviews commonly conclude that rodents show behavioral changes at low blood lead levels, most of the evidence for such effects pertains to lead levels of the animals at the time of testing (i.e., chronically exposed to lead). It has proven very difficult to demonstrate ***lasting*** cognitive deficits following a short period of early exposure to low or even moderately elevated blood lead levels in animal models (e.g., Ferguson et al. 1998). The demonstration of lasting

cognitive deficits (i.e., when tested long past the exposure period) was essential for the success of the current study.

Despite the transiently high tissue lead levels seen in the High Pb animals during the final week of exposure, none of the animals exhibited any signs of overt toxicity throughout the study (changes in grooming, fur condition, interactions with littermates, etc.), and could not be distinguished from Controls throughout the 6-8 months of daily handling. These findings are consistent with prior evidence that the blood lead levels required to produce overt toxicity (i.e., malaise, coma, convulsions, death) or lasting neurobehavioral effects (i.e., effects that last well beyond the period of exposure) are higher in rats than in humans (see Discussion section of published report). For this reason, it may not be appropriate to directly extrapolate effects produced by specific blood lead levels from rats to humans. Moreover, for the goals of the present study, it is not important that the specific blood lead levels needed to produce lasting behavioral effects are the same in the rat and human, only that the same constellation of effects are produced (e.g., the pattern of neurobehavioral effects; presence or absence of overt toxicity), thereby allowing a test of the hypothesis that these effects can be ameliorated by succimer treatment. (Further discussion of this issue is provided in the Discussion section of the published report.)

Tissue lead analyses: On PND 52 (end of chelation therapy) one animal from each litter was euthanized for analysis of blood and brain lead levels. A 2 - 3 ml sample of whole blood was collected into a polypropylene syringe via cardiac puncture from surgically exposed hearts of anesthetized animals, and deposited into low-lead vacutainers specified for trace metal analyses (#367734, Becton Dickson, Research Triangle Park, NC). Whole brain was removed using acid-

washed stainless-steel dissecting tools, rinsed with Milli-Q water and deposited into polypropylene storage containers. All tissue sampling was conducted using trace metal-clean procedures, as detailed elsewhere (Smith et al. 1998). All samples were stored frozen. Blood and brain lead levels were measured at the University of California, Santa Cruz, using a Finnegan Element inductively coupled plasma (ICP) - high resolution mass spectrometer in multi-isotope counting mode, measuring masses ^{208}Pb and ^{209}Bi , with ^{209}Bi used as an internal standard (Smith et al. 2000). This ICPMS methodology has been demonstrated to yield a measurement precision of approximately ± 1-2% for sample lead concentrations > 0.05 ppb. The analytical detection limit was 0.01 ppb.

Dependent measures: For the Visual Discrimination Task, an analysis was conducted on the average percent correct for the first six sessions on the task; after this point, the groups converged because most animals in all groups had mastered the task by this time. Percent correct was calculated as the number of correct trials divided by the total number of response trials in that session, multiplied by 100 to yield a percentage. A "response trial" was defined as a trial on which the rat entered the testing alcove at trial onset. This type of analysis permitted an evaluation of different patterns of learning, contrary to using total trials- or errors-to-criterion as the dependent measure. Animals reaching criterion in fewer than six sessions were given a score of 85% correct for the post-criterial sessions, based on the average asymptotic performance level achieved in this task (e.g., Gendle et al. 2003; Morgan et al. 2001).

For each of the attention tasks, an analysis was conducted for each of the three error types (omission errors, premature responses, and inaccurate responses), converted to a percentage. Prior to analysis, an average was calculated for each rat for each testing condition (such as Delay

and Session-block). For each of these error types, the dependent measure was calculated as the number of errors in that condition divided by the number of response trials in that condition, multiplied by 100 to yield a percentage. The GLMM analyses were conducted on these means.

Statistical procedures: The various performance measures were analyzed using a generalized linear mixed model (GLMM) which correctly handled the repeated measures on each animal (Wolfinger and O'Connell 1993). The distribution of model residuals was specified as binomial or normal depending on the type of outcome. Separate analyses were conducted for each of the three lead exposure levels, corresponding to the *a priori* hypotheses: (1) High Pb, High-Pb-succ, and Control (no lead exposure or succimer); (2) Mod Pb, Mod Pb-succ, and Control; and (3) Succimer-only (no lead + succimer) and Control. As appropriate for the task, the models included: Treatment, pre-cue delay (Delay), cue duration (Duration), Session-block (day on task, grouped into blocks for analysis), Trial-block (the 200 trials per session, divided into 3 blocks, each with 66 or 67 trials), outcome of the previous trial (correct or incorrect, termed "Prev"), Distraction condition (described below), and all relevant interaction terms. The dependent measure used to assess motivation (#RT/session length) was analyzed by analysis of variance, using a Tukey multiple comparison procedure to adjust the significance level.

Restricted feeding regimen: All animals were initially given 18 g food/day, comprised by the Noyes pellet rewards plus additional chow given immediately after the daily testing session. On non-testing days (Saturdays), animals were given 5 h to eat their daily allotment. For the majority of animals, the initial food allotment (18 grams) was not altered throughout behavioral testing. However, for some animals (across all groups), this amount of food seemed to be

excessive with respect to maintaining motivation throughout each daily test session, as indicated by not completing the target 200 response trials per session for 3 or more consecutive sessions or more than 3 sessions over a 10 day period. For these rats, food intake was reduced by a gram. If the pattern continued, the allotment was reduced by an additional gram until sufficient motivation was established (i.e., they completed between 175 and 200 trials per session). For a few other animals (one or two animals in total, across all groups), this initial 18 gram allotment appeared to be insufficient, as indicated by sub-normal weight gain. For these animals, the daily food allotment was increased by a gram or two. The goal was to ensure equal hunger at the time of daily testing. These manipulations in food intake were made by individuals blind to the treatment conditions of the animals.

Although the daily food needs varied somewhat across animals within a group (presumably reflecting differences in metabolic rate), they did not differ systematically across groups (see Results section of published article).

Results

Sustained Attention Task (some additional details that could not be presented in the published report due to space constraints)

Percent Omission errors: An omission error was tallied when a rat entered the testing alcove but failed to respond within 15 s of trial onset, indicative of missing the cue. Prior studies in our lab have revealed that one factor that increases omission error rate is committing an error on the previous trial. Because committing an error increases the rate of both omission errors and nontrials on the subsequent trial, it was deemed optimal to include nontrials as an “event” in the

analysis of omission errors to better gauge reactivity to errors, an area that has proven sensitive to lead in our prior studies (e.g., Morgan et al. 2001). Because nontrial rates were quite low overall, and virtually nonexistent under certain conditions (e.g., for trials following a correct response), it was not possible to analyze them separately with complex models. For simplicity, this dependent measure will generally be referred to as omission error rate because the results were driven predominantly by omission errors.

In addition to the results presented in the published report, the analysis of the Mod Pb, Mod Pb-succ, and Control rats revealed a trend for an adverse effect of succimer in the Mod Pb group (described below). Because this effect was only a statistical trend, and because this was the only endpoint for which succimer impaired performance of lead-exposed rats in the present four tasks, it should be viewed as tentative, and in need of replication.

Significant interactions of Treatment and Trial-block [$F_{(4,105)} = 2.54$; $p = 0.04$], as well as Treatment, Trial-block, and PREV [$F_{(4,881)} = 2.81$; $p = 0.02$]. This 3-way interaction reflects two different effects: (1) For trials following a correct response, the Mod Pb group tended to commit slightly more omission errors than Controls, specifically in trial-block 1 (first 66 trials) ($p = 0.06$), not later in the session (means for the Control and Mod-Pb groups were 3.0 % and 4.1%, respectively). This pattern suggests a slight impairment in settling-in to the task at the beginning of each testing session. The Mod Pb-succ group was intermediate to the other two groups and not significantly different from either one (mean = 3.6%). (2) Second, for trials following an error, the rate of increase of omission errors across the session was greater for the succimer-treated Mod Pb rats than for the Controls ($p = 0.02$) and the Mod Pb rats ($p = 0.01$), with the result that by the third trial-block, the succimer-treated Mod-Pb rats tended to commit more omission errors than the Controls ($p = .07$). Inspection of the raw data indicated that this

significant group difference was driven by the Mod Pb-succ group having the largest proportion of rats that would often commit long strings of nontrials following an error, specifically in the final block of trials in each test session. This pattern suggests low persistence in the face of an error specifically at the end of each session, when attention and motivation were waning.

Body weight and performance:

As discussed in the published article, a comparison of the body weights of the six groups on PND 53, the day after cessation of succimer treatment, revealed that the High-Pb and High Pb-succ groups weighed ≈ 6% less than the Control group ($p < 0.05$). None of the other groups differed from Controls. The slightly smaller size of the High Pb rats likely reflects a specific lead effect such as a decrease in growth hormone during the lead exposure period (e.g., Ronis et al. 1998), rather than overt toxicity, since there were no differences in the appearance or activity of the animals across any of the lead exposure groups throughout the the 6-8 months of daily handling. All appeared healthy and well-groomed throughout the study.

Several lines of evidence argue against the idea that the small body weight deficit of the High Pb rats contributed, directly or indirectly, to the behavioral effects presented above. First, the High Pb rats did not differ from the Controls in motivation (see Results section of published article). Second, succimer treatment alleviated cognitive deficits in the High Pb rats without mitigating this body weight effect. And finally, the majority of instances of impairment seen in the High Pb rats were limited to trials with specific characteristics (e.g., those that followed an error, had a specific cue duration, pre-cue delay, etc), arguing against mediation by body weight differences.

REFERENCES

Alber SA, Strupp BJ. 1996. An in-depth analysis of lead effects in a delayed spatial alternation task: Assessment of mnemonic effects, side bias and proactive interference. Neurotoxicol Teratol 18:3-15.

Ferguson SA, Holson RR, Gazzara RA, Siitonen PH. 1998. Minimal behavioral effects from moderate postnatal lead treatment in rats. Neurotoxicol Teratol. 20:637-43.

Garavan H, Morgan RE, Levitsky DA, Hermer-Vazquez L, Strupp BJ. 2000. Enduring effects of early lead exposure. evidence for a specific deficit in associative ability. Neurotoxicol Teratol 22:151-164.

Gendle MH, Strawderman M, Mactutus CF, Levitsky DA, Booze RM, Strupp BJ. 2003. Alterations in reactivity to errors and sustained attention in an animal model of prenatal cocaine exposure. Dev Brain Res 147: 85-96.

Hilson JA, Strupp BJ. 1996. Analyses of response patterns clarify lead effects in olfactory reversal and extradimensional shift tasks: assessment of inhibitory control, associative ability, and memory. Behav Neurosci 111:532-542.

Morgan RE, Garavan H, Smith EG, Driscoll LL, Levitsky DA, Strupp BJ. 2001. Early lead exposure produces lasting changes in sustained attention, response initiation, and reactivity to errors. Neurotoxicol Teratol 23:519-531.

Ronis MJ, Badger TM, Shema SJ, Roberson PK, Templer L, Ringer D, et al. 1998. Endocrine mechanisms underlying the growth effects of developmental lead exposure in the rat. J Toxicol Environ Health A 54:101-120.

Smith D, Bayer L, Strupp BJ. 1998. Efficacy of succimer chelation for reducing brain Pb levels in a rodent model. Environ Res 78:168-176.

Smith DR, Woolard D, Luck ML, Laughlin NK. 2000. Succimer and the reduction of tissue lead in juvenile monkeys. Toxicol Appl Pharmacol 166:230-240.

Stangle DE, Strawderman MS, Smith D, Kuypers M, Strupp BJ. 2004. Reductions in blood lead overestimate reductions in brain lead after repeated succimer regimens in a rodent model of childhood lead exposure. Environ Health Perspect 112:302-308.

Wolfinger R, O'Connell M. 1993. Generalized linear mixed models: a pseudo-likelihood approach. J Stat Comp Simul 48:233-244.
